# Dynamic Response and Energy Absorption of Lattice Sandwich Composite Structures Under Underwater Explosive Load

**DOI:** 10.3390/ma18061317

**Published:** 2025-03-17

**Authors:** Xiaolong Zhang, Shengjie Sun, Xiao Kang, Zhixin Huang, Ying Li

**Affiliations:** 1 School of Naval Architecture, Ocean and Energy Power Engineering, Wuhan University of Technology, Wuhan 430064, China; 2 Institute of Advanced Structure Technology, Beijing Institute of Technology, Beijing 100081, China

**Keywords:** lattice structure, underwater explosion, dynamic response, energy absorption, fluid–structure interaction

## Abstract

This study investigates the underwater explosion resistance of aluminum alloy octet-truss lattice sandwich structures using shock tube experiments and LS-DYNA simulations. A systematic analysis reveals key mechanisms influencing protective performance. The sandwich configuration mitigates back plate displacement through quadrilateral inward deformation, exhibiting phased deformation responses between face plates and back plates mediated by lattice interactions. Increasing the lattice relative density from 0.1 to 0.3 reduces maximum back plate displacement by 22.2%. While increasing the target plate thickness to 1.5 mm reduces displacement by 47.6%, it also decreases energy absorption efficiency by 20% due to limited plastic deformation. Fluid–structure interaction simulations correlate well with 3D-DIC deformation measurements. The experimental results demonstrate the exceptional impact energy absorption capacity of the octet-truss lattice and highlight the importance of stiffness-matching strategies for enhanced energy dissipation. These findings provide valuable insights for optimizing the design of underwater protection structures.

## 1. Introduction

The survivability of naval vessels and critical maritime security assets is predominantly determined by their shock resistance and blast resistance capabilities [1]. Underwater blast-induced shock waves exhibit higher propagation velocities in aquatic environments compared to atmospheric conditions, consequently imposing more substantial structural loads on marine vessels than aerial explosions [2,3]. Experimental and numerical investigations of sandwich structures consistently demonstrate that metal-core configurations possess enhanced blast resistance relative to their monolithic counterparts with equivalent areal density, attributable to their optimized energy absorption mechanisms [4,5,6].

Investigations of impact-resistant structures have integrated experimental and computational approaches to systematically characterize dynamic response characteristics and failure mechanisms under blast loading scenarios [7,8,9]. Wadley et al. found that multilayer pyramid lattice structures exhibit a distinct stress plateauing at 60% compressive strain during underwater blast events, thereby attenuating transmitted impulse through controlled energy dissipation mechanisms [10]. Huang et al. demonstrated that controlling the impact velocity can effectively replicate underwater blast pressure profiles, including peak pressure magnitude and temporal attenuation profiles, while their experimental data revealed that foam-reinforced composite lattice architectures demonstrate 32% greater energy absorption efficiency compared to carbon fiber-reinforced polymer (CFRP) laminates under equivalent impulse conditions [11]. Liu et al. developed Composite Tube-Reinforced Hyperelastic Porous Polyurethane (CRHPP) structures showing 407% enhanced energy absorption compared to standard porous polyurethane. The dynamic response and failure mechanisms of CRHPP under underwater explosive loading were systematically investigated through combined experimental and computational approaches. The results showed that composite tube incorporation yielded a 2.8-fold stiffness enhancement and 4.3× energy dissipation capacity improvement in hyperelastic PU matrices through multi-scale energy dissipation mechanisms [12]. Huang et al. revealed that core height in aluminum corrugated sandwich panels predominantly governs the dynamic response rate rather than dictating global deformation patterns under blast loading. The analysis systematically categorized the structural evolution into three distinct phases: (1) initial elastic regime, (2) dynamic plastic hinge formation, and (3) progressive plastic collapse. Furthermore, they identified permanent back-face deformation as a critical parameter in blast-resistant performance metrics, correlating directly with post-blast structural integrity [13]. Zhang et al.’s experimental investigations revealed axisymmetric deformation propagation in spherical shells, initiating from peripheral regions and converging toward the geometric center with maximum displacement magnitude. Shell curvature-induced wave convergence phenomena significantly modulated reflected wave dynamics. Quantitative analysis demonstrated a velocity-dependent amplification mechanism where increased central deformation rates correlated with enhanced residual displacement under equivalent loading conditions. Furthermore, nonlinear dependencies between post-impact residual displacement and geometric parameters (thickness-to-curvature ratio) were quantitatively verified through systematic impact testing [14]. A finite element analysis of CFRP-lattice sandwich structures under shock wave loading by He et al. identified three failure modes governed by shock wave intensity, core configuration, and face sheet parameters. Structural stiffness and impulse influenced the observed failure modes. Core failure correlated with shock wave intensity [15].

The exceptional specific strength and energy absorption of lattice truss cores motivate their widespread use in sandwich structures for diverse engineering applications [16]. The tension-dominated microstructure of octet-truss lattices yields high elastic modulus and yield strength, making them suitable for impact protection applications [17]. Ling et al. demonstrated that the macroscopic properties of composites are fundamentally determined by the matrix material, with high-ductility matrices enabling superior energy absorption via extensive plastic deformation [18]. Experimental and simulation results (Weeks et al.) confirm isotropic stiffness and efficient energy dissipation in these low-density metamaterials [19]. 

This study investigates the dynamic response and energy absorption of aluminum alloy-lattice sandwich structures subjected to underwater shock loading. Shock tube simulations inform equivalent impact tests and validate finite element models. Numerical analyses elucidate component interactions, deformation mechanisms, and energy absorption efficiency. The influence of relative density and face plate thickness on structural performance under varying shock loads provides insights for naval anti-explosion design.

## 2. Experimental Setup

### 2.1. Experimental Devices

**Figure 1:**
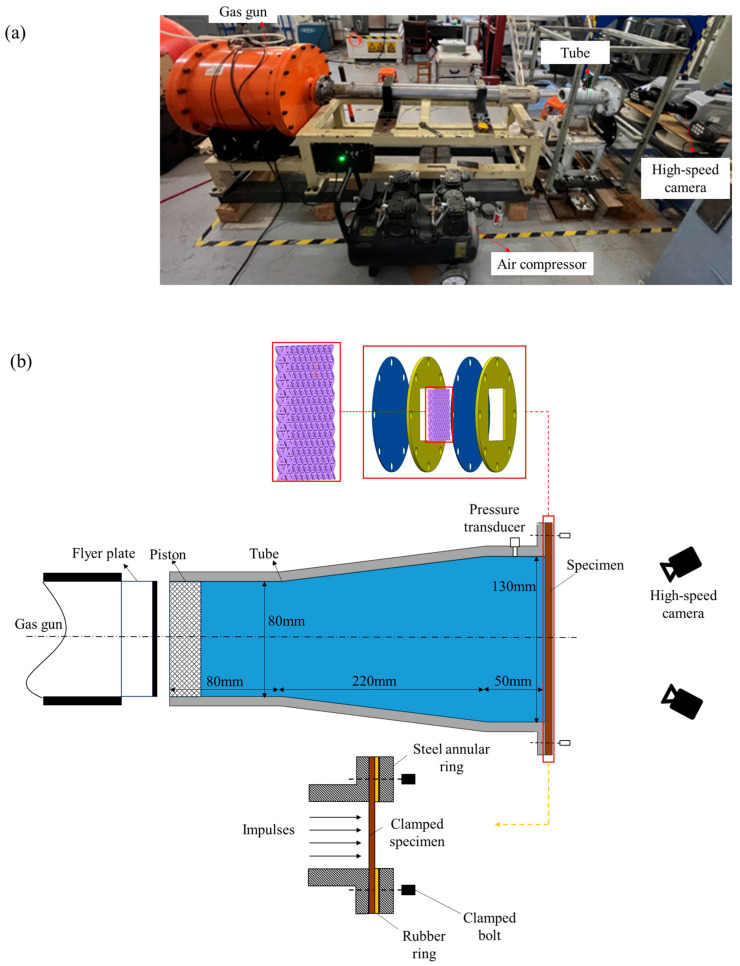
Underwater shock loading system. (**a**) Photo. (**b**) Schematic drawing.

Figure 1 depicts the experimental setup for investigating the dynamic response of metallic lattice sandwich panels to underwater pulse loading. A light gas gun-driven flyer plate impacts a shock tube piston, generating planar pressure pulses that simulate underwater explosion shock waves. The apparatus comprises a light gas gun, an air compressor, a shock tube, an underwater pressure measurement system, a target plate, and a 3D-DIC system with two high-speed cameras.

The Q345 steel shock tube consists of three sections: a rear segment (80 mm × 80 mm), a transition segment (220 mm long), and a front segment (50 mm × 130 mm), all with a 15 mm wall thickness. O-ring seals ensure watertightness at the piston and panel interfaces. Underwater pressure is measured using a 2004L-50 IEPE sensor produced by Kedong Electronics, Yangzhou China (50 mV/MPa sensitivity) and a 8302 acquisition instrument produced by Donghua, Jiangsu China (1 MHz sampling rate). A CSI 3D-DIC system produced by LTY, Beijing China with two Phantom V1212 high-speed cameras produced by Vision Research, Wayne, New Jersey U.S (20,000 fps) captures target plate strain and deformation. Consistent camera angles ensure reliable results across all tests.

### 2.2. Target Plate Construction

**Figure 2:**
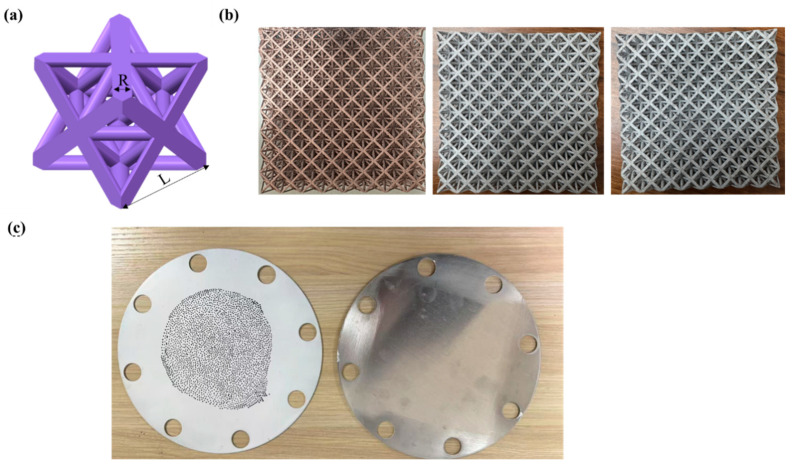
Target plate and lattice diagram: (**a**) octet truss lattice; (**b**) lattice core; (**c**) target plate.

The target comprises two 1100-O aluminum plates (102 mm radius, 1 mm thick). The back plate is painted white and marked with positioning dots for 3D-DIC deformation measurement. Three 1100-O aluminum octet-truss lattice core configurations with varying relative densities are selected. Each lattice unit cell has a length (L) of 10 mm, with strut radius (R) determined by the relative density (lattice volume/occupied spatial volume). Figure 2 shows the target plate and lattice structures, with dimensions detailed in Table 1. An approximate analytical formula describes the relative density of octet-truss lattices (cylindrical struts, unit length L, and radius R) at low values [17].

(1) $$\overset{-}{\rho }=6\sqrt{2}\pi (\frac{R}{L}{)}^{2}$$

The lattice core and target plates are clamped between two 10 mm thick circular flanges (yellow components in Figure 1b) with central 80 mm square slots. Eight M16 bolts secure the assembly to the shock tube. Both the loading area and lattice-filled region are 80 mm squares. The lattice and target plates are not bonded, allowing some relative motion during testing.

### 2.3. Experimental Operation

**Figure 3:**
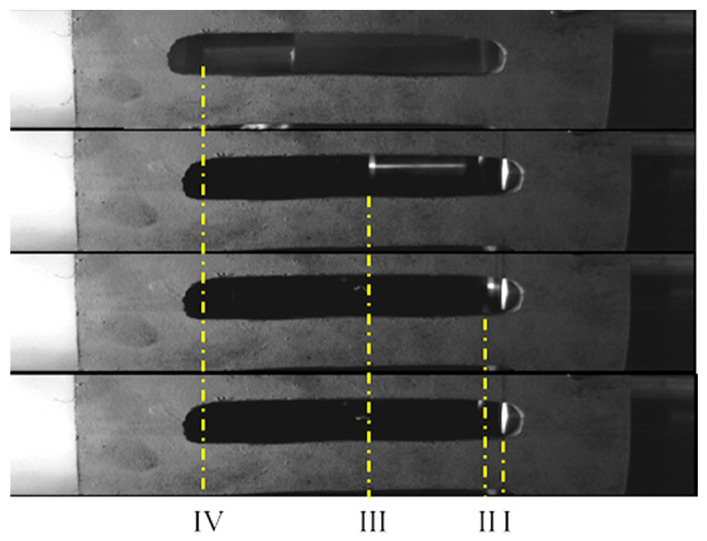
Flyer motion process: (I) launch tube exit; (II) full launch tube exit; (III) timed trajectory; (IV) initial piston impact.

Flyer plate velocity was controlled by adjusting gas chamber pressure and loading distance. High-speed cameras captured flyer plate motion (Figure 3) across four zones: I (launch tube exit), II (full launch tube exit), III (timed trajectory), and IV (initial piston impact). Timing the flyer plate’s passage through Zones II–IV allowed velocity calculation. Pretesting revealed poor repeatability with low flyer speeds and potential danger at high chamber pressures. Consequently, a 41.5 m/s flyer speed was selected. Tests confirmed this speed with a 50 cm loading distance and 0.3 MPa of chamber pressure.

Three lattice relative densities (0.1, 0.2, and 0.3), corresponding to Cases 1–3, were tested under a 41.5 m/s flyer plate velocity. Underwater shock wave pressure profiles were recorded by pressure sensors at the shock tube’s rear end, and back plate dynamic responses were captured by the 3D-DIC system. Table 2 summarizes the detailed results.

## 3. Finite Element Simulation

### 3.1. Finite Element Model

**Figure 4:**
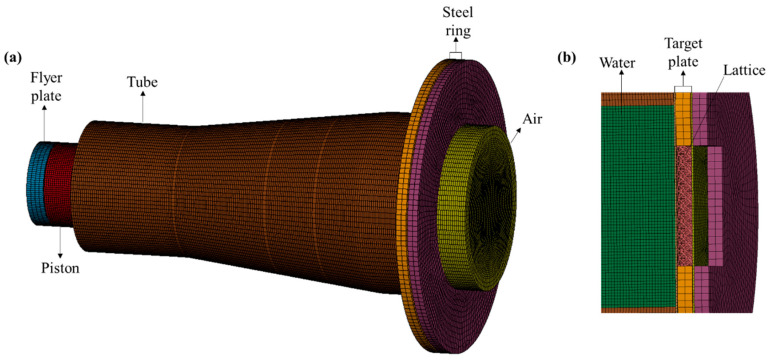
Finite element model: (**a**) the whole model; (**b**) cut view.

The finite element model was developed using the commercial software LS-DYNA version 4.10 [20], as illustrated in Figure 4. The full model included the target plate, lattice core, shock tube, flanges, piston, flyer plate, air, and water. The Arbitrary Lagrange–Euler (ALE) algorithm, a validated method for fluid–structure interaction and underwater shock simulations, modeled the air and water domains [14,21]. Solid elements discretized all other components. Fixed boundary conditions replicated experimental constraints on the shock tube and flanges. Component interactions used the AUTOMATIC_SURFACE_TO_SURFACE contact algorithm, while self-contact (CONTACT_AUTOMATIC_SINGLE_SURFACE) simulated lattice densification. CONSTRAINED_LAGRANGE_IN_SOLID handled fluid–structure coupling. Table 3 summarizes key solver settings to prevent mesh distortion. An initial velocity was assigned to the flyer plate to impact the piston and generate planar shock waves.

### 3.2. Material Constitutive Model

**Figure 5:**
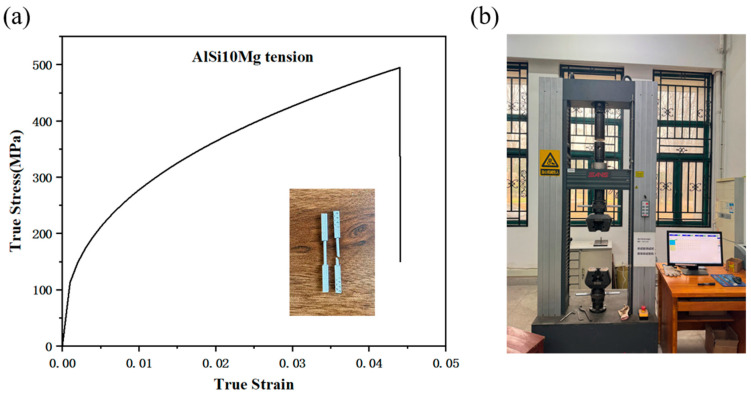
(**a**) True stress–strain curve for 1100-O aluminum alloy and (**b**) MTS 5205 universal testing machines.

Quasi-static tensile tests (Figure 5a) provided the true stress–strain curves for the 1100-O aluminum alloy. The tensile test used an CMT 5205 machine produced by MTS, Eden Prairie, Minnesota U.S (Figure 5b) following the GB/T 16491-1996 standard and GB/T 228.1-2021 [22] test standard at a rate of 1 mm/min. The parameters of the MAT_POWER_LAW_PLASTICITY constitutive model, calibrated to these data, are listed in Table 4. Negligible deformations in the 4340 alloy steel piston and Q345 steel fixtures were modeled using MAT_PLASTIC_KINEMATIC (parameters in Table 5).

Fresh water was used to simulate the water medium, with its state described by the Gruneisen equation of state. The pressure (*P*) of the water under this equation of state is:

(2) $$P=\frac{\rho_{0}C^{2}u\left[1+\left(1-\frac{\gamma_{0}}{2}\right)u-\frac{a}{2}u^{2}\right]}{{\left[1-\left(S_{1}-1\right)u-S_{2}\frac{u^{2}}{u+1}-S_{3}\frac{u^{3}}{{\left(u+1\right)}^{2}}\right]}^{2}}+\left(\gamma_{0}+au\right)E$$

where $\rho_{0}$ is the density of water; u represents the rate of volume change; ***C*** is the speed of sound traveling through water; a is the Gruneisen parameter; $\gamma_{0}$ is a first-order correction factor for parameter a; $S_{1}$, $S_{2}$, and $S_{3}$ are the dimensionless coefficient of the velocity of the shock wave versus the slope of the fluid mass velocity curve; and *E* is the initial internal energy per unit volume of the modeled material. Specific parameters are shown in Table 6.

A polynomial equation of state is used to describe the pressure of air medium, the keyword used in Ls-Dyna version 4.10 is EOS_LINEAR_POLYNOMIAL, and the specific expression is:

(3) $$P=C_{0}+C_{1}\mu +C_{2}\mu^{2}+C_{3}+(C_{4}+C_{5}\mu +C_{6}\mu^{2})\varepsilon$$

where μ is the relative volume; ε is the internal energy per unit volume; and $C_{0}$ to $C_{6}$ are the fitting coefficients; for ideal gases, $C_{4}$ and $C_{5}$ are 0.4, and 0 for the rest. Specific parameters are shown in Table 7.

## 4. Results and Discussion

### 4.1. Dynamic Impact Response of Sandwich Structures

**Figure 6:**
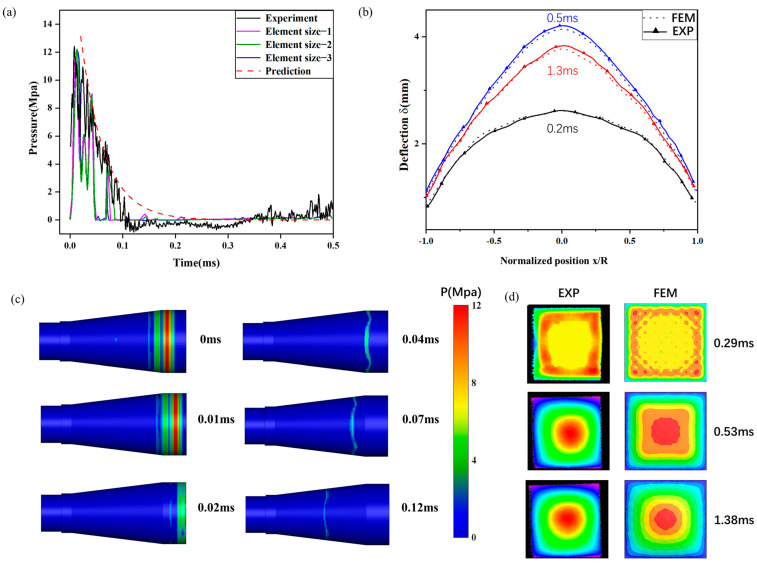
Validation of experiments: (**a**) pressure history curve, (**b**) comparison of back plate deflection at different times, (**c**) shock wave propagation process, and (**d**) deformation of back plate.

Due to test condition limitations, observing the dynamic panel and core layer response was difficult. Therefore, the experimental results were compared to those of the flying lattice at 41.5 m/s and a relative density of 0.1. The classical Taylor analytical equation described the excitation wave attenuation in the fluid at a fixed position [23]:

(4) $$P(t)=P_{m}e^{-\frac{t}{\theta }}$$

where $P_{m}$ denotes the peak pressure and θ denotes the decay time; the pressure profile at the shock tube’s tail end was obtained using this equation. A mesh convergence study using 1 mm, 2 mm, and 3 mm fluid domain mesh sizes produced the shock wave pressure curves in Figure 6a. Comparing these with experimental data yielded peak pressure errors of 7.71%, 1.55%, and 2.28%, and pulse width errors of 13.42%, 5.48%, and 14.75%, respectively. The computation times were 4.8 h, 2.9 h, and 2.5 h. A 2 mm mesh size was chosen for the subsequent Eulerian domain, balancing accuracy and computational cost.

Figure 6c depicts underwater shock wave transmission and initial reflection in the shock tube. The pressure measurement time is defined as the initial moment. The shock wave maintains a planar shape before impacting the target plate. The tapered shock tube design mitigates reflected wave interference, preserving a regular shock wave shape during reverse propagation.

Figure 6b compares numerical and experimental back plate deformation results, showing center point displacement errors of 1.23%, 1.31%, and 1.43% (all below 5%). This validates the underwater shock tube numerical model’s efficacy in replicating the experimental setup.

**Figure 7:**
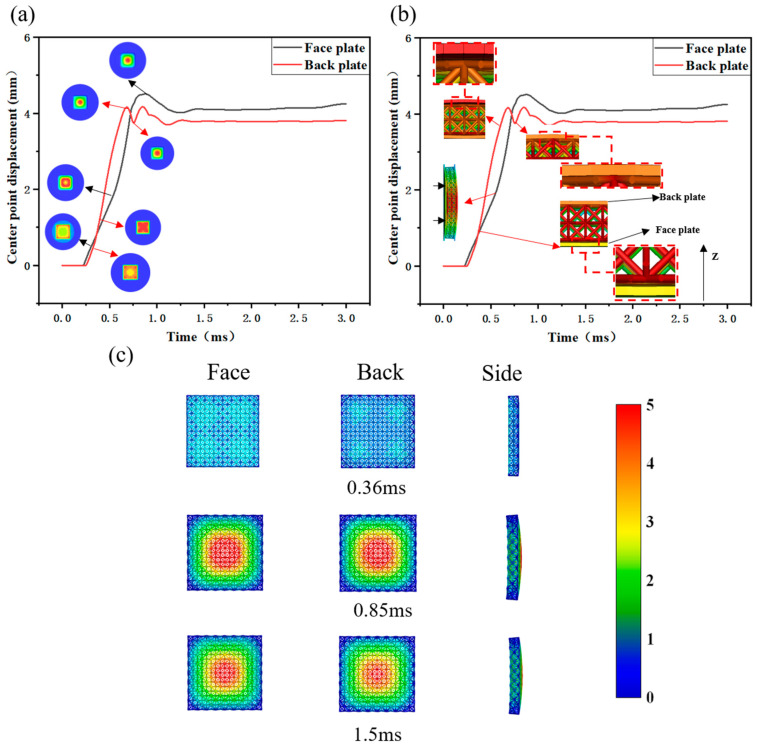
Midpoint displacement curves and dynamic response of lattice sandwich panels under underwater shock wave: (**a**) dynamic response and deformation nephogram of face and back plate; (**b**) interaction between lattice and target plate; (**c**) dynamic response of lattice.

Figure 7 illustrates the midpoint displacement curve and dynamic response under an underwater shock wave. The sandwich plate’s face plate deforms initially, followed by lattice structure deformation influenced by the face plate, which in turn drives back plate deformation. The lattice’s presence significantly reduces the face plate deformation rate compared to the back plate. Over time, increasing target plate deformation energy requirements coupled with diminishing shock wave energy cause deformation rates to decrease. The reflected shock wave propagates in the opposite direction. At this point, the sandwich plate’s stored mechanical energy exceeds the deformation energy requirement, leading to rebound and, ultimately, complete plastic deformation after the interaction between the face plate, lattice structure, and back plate.

Figure 7a shows the dynamic response of both face and back plates, revealing distinct deformation modes due to the lattice. Initially, the face plate deforms around the loading area post-impact, gradually shrinking towards the center over time. The face plate’s deformation rate increases significantly upon reaching the center. The back plate, influenced by the lattice, deforms initially at the corners, progressively shrinking towards the center, forming an “X” shape before finally converging to the center. The back plate’s maximum central displacement is 4.16 mm, with a final deformation displacement of 3.8 mm. The face plate’s maximum central displacement reaches 4.51 mm, settling at a final deformation displacement of 4.25 mm.

Figure 7b illustrates the interaction between the lattice and target plate, offering insights into the face and back plate fluctuations during rebound. As the face plate deformation progresses from the periphery to the center, a brief separation occurs between the face plate and lattice at the center. The lattice and back plate continue moving in the positive *Z*-axis direction. Due to the lattice’s higher stiffness compared to the target plate, the back plate separates from the lattice, reaching maximum displacement first. It then rebounds due to stored mechanical energy. After a period, it re-establishes contact with the lattice. Since the lattice is still deforming at this point, the back plate’s displacement increases again before rebounding once more upon reaching maximum displacement, finally settling into plastic deformation.

Figure 7c shows the lattice’s deformation process in three directions. At 0.34 ms, the shock wave directly impacts the face plate, compressing and deforming the lattice structure. Deformation on the lattice’s front side concentrates primarily at the corners, while the back side remains nearly intact, indicating that the shock wave energy failed in fully transferring to the back plate. Maximum lattice deformation occurs at 0.85 ms, with the largest displacement at the center of the lattice’s front side. The back side also shows significant displacement, especially in the area opposite the front side. By 1.5 ms, the structure’s deformation stabilizes, and the lattice rebounds due to its stored mechanical energy. The lattice’s side view reveals significant plastic deformation after loading, demonstrating its good energy absorption capacity.

### 4.2. Effect of Relative Density on Deformation and Energy Absorption Properties

The rod lattice’s relative density significantly influences the structure’s stiffness and strength. Increased relative density, achieved through larger rod diameters, enhances compressive capacity. However, this increased stiffness reduces the lattice’s ability to absorb energy through plastic strain, making it more prone to localized fracture [24]. Therefore, it is crucial to investigate the effects of both relative density and projectile velocity on the protective structure’s performance.

**Figure 8:**
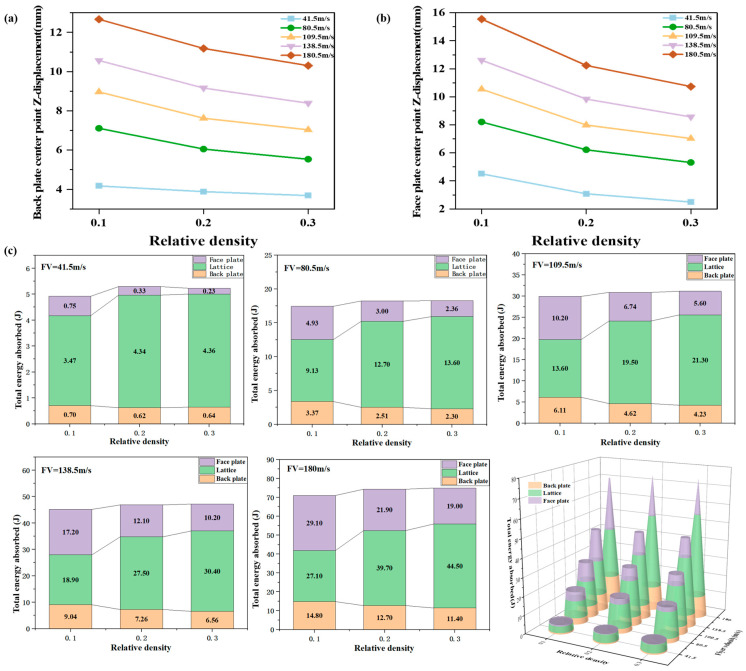
Effect of relative density and flyer velocity on sandwich protection structure: (**a**) midpoint displacement of face plate; (**b**) midpoint displacement of back plate; (**c**) energy absorption ratio.

Figure 8a and Figure 8b illustrate the displacement variations at the center of the face and back plates as a function of relative density and projectile velocity, respectively. A notable displacement change correlated with relative density is observed at 80.5 m/s. With relative densities of 0.1 and 0.3, the maximum back plate displacements are 7.11 mm and 5.53 mm, respectively, representing a 22.22% decrease. At lower projectile velocities, the figures suggest that the influence of lattice relative density is less pronounced on the back plate’s peak displacement but more significant on the face plate. This is attributed to the lower impact load of the shock wave at lower velocities. Consequently, the face plate and lattice structure absorb a greater proportion of the energy through plastic deformation, transmitting a smaller load fraction to the back plate. As projectile velocity increases, the face plate displacement in lower-density lattices exhibits a marked upward trend. At a projectile velocity of 180 m/s, face plate damage occurs. This is likely due to the reduced overall stiffness of the lattice at this velocity, resulting in greater deformation and an inability to adequately support the face plate against the impact load. Thus, the lattice structure functions primarily as a protective layer for the face plate. At a constant velocity, a higher lattice relative density correlates with reduced face plate deformation. Back plate deformation, however, is influenced synergistically by the deformation of both the face plate and the lattice structure.

Figure 8c illustrates the influence of relative density on energy absorption by each component of the sandwich panel at various projectile velocities. At lower projectile velocities, the shock wave impact load is minimal, and the protective structure’s energy absorption increases with relative density. As lattice relative density increases, the lattice provides greater support to the face plate, consequently reducing the deformation of both the face and back plates, and thus decreasing the proportion of energy absorbed through plastic deformation. Concurrently, the proportion of energy absorbed by the lattice increases with increasing projectile velocity. This is attributed to the higher overall stiffness of the lattice compared to the face and back plates, resulting in greater energy absorption through plastic deformation. Furthermore, as projectile velocity increases, the energy absorbed by both the entire structure and the lattice increases. At lower loads, a lattice with a relative density of 0.2 sufficiently supports the face plate to withstand the impact. Further increases in relative density, under these conditions, limit further lattice deformation, causing the lattice to transition from an energy-absorbing component to a load-transferring component. Therefore, a higher lattice relative density does not necessarily equate to improved energy absorption; the optimal relative density is dependent on the magnitude of the shock wave load. Under low-load conditions with excessively high lattice relative density, a significant portion of the energy is transmitted through the lattice and target plate to subsequent structural elements.

### 4.3. Effect of Plate Thickness on Deformation and Energy Absorption Properties

As discussed previously, the relative density significantly influences the protective performance of the structure, with contributions from both the target plate and the lattice. Therefore, with the lattice relative density held constant at 0.1, target plate thickness is now considered as a variable to analyze the deformation behavior of the face and back plates under various projectile velocities.

**Figure 9:**
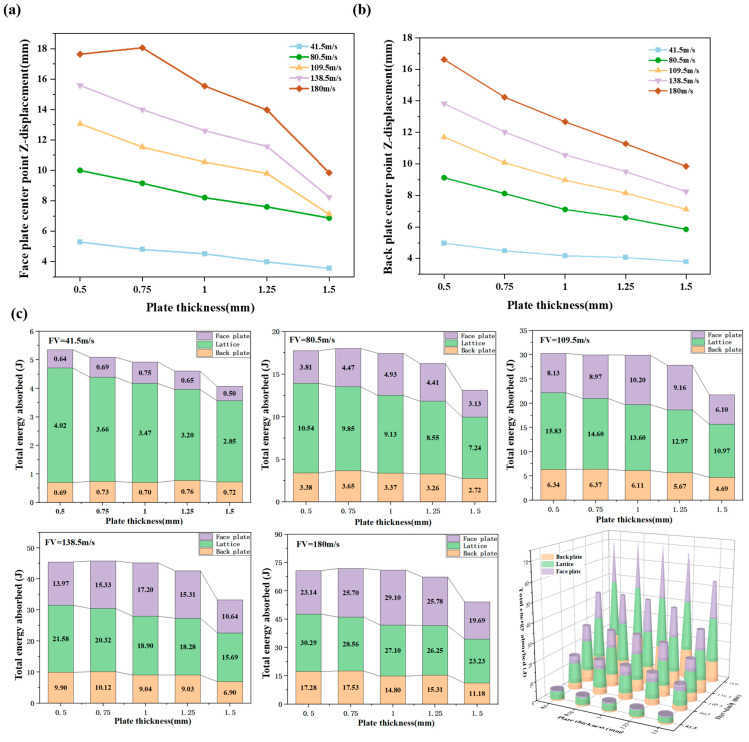
Effect of target plate thickness and flyer velocity variation on sandwich protection structure: (**a**) midpoint displacement of face plate; (**b**) midpoint displacement of back plate; (**c**) energy absorption ratio.

Figure 9a,b present the face and back plate deformation at various projectile velocities with varying target plate thicknesses. At a projectile velocity of 180 m/s, the center point displacement of the back plate is 16.62 mm with a 0.5 mm thick target plate and 9.85 mm with a 1.5 mm thick target plate, representing a 40.7% reduction in maximum displacement. The results also indicate a significant influence of target plate thickness on the peak displacement of the face plate.

At higher projectile velocities, a substantial decrease in peak face plate displacement is observed when the target plate thickness increases from 1.25 mm to 1.5 mm. Conversely, the peak back plate displacement exhibits a less pronounced, more linear response to changes in target plate thickness. This is primarily because the shock wave load acts directly on the face plate. Increased face plate thickness corresponds to increased strength, resulting in reduced peak displacement under the same shock wave load.

The back plate, however, is primarily influenced by the load transferred through the deformation of the face plate and lattice structure, and thus exhibits a less sensitive response to target plate thickness variations. Figure 9c presents the influence of target plate thickness variation on the energy absorption of each structural component at different projectile velocities. At lower projectile velocities, the overall energy absorption of the structure decreases with increasing target plate thickness. Concurrently, the energy absorption ratios of the lattice and face plate decrease, while the back plate’s energy absorption ratio increases.

As projectile velocity increases, the energy absorption initially increases and then decreases. At a projectile velocity of 180 m/s, the total energy absorption of the structure with a 1.5 mm thick target plate is 20% lower than that with a 0.5 mm thick target plate. Simultaneously, the proportion of energy absorbed by the lattice decreases. The energy absorption ratio of the face plate mirrors the trend of overall energy absorption, increasing initially and subsequently decreasing. This behavior is primarily attributed to the increased stiffness of the face plate with increasing thickness.

Lower pressure load is insufficient to induce significant deformation, resulting in most of the energy being transferred through the face plate and lattice to the back plate. As the load increases, it becomes sufficient to cause plastic deformation in the thinner target plates, leading to an increased energy absorption ratio. These observations suggest an interdependent relationship between target plate thickness and lattice relative density.

The component with higher stiffness (either the face plate or the lattice) plays a dominant role in supporting the structure. However, excessive stiffness hinders plastic deformation, impacting energy absorption. Therefore, the optimal target plate thickness and lattice relative density should be determined based on the specific load magnitude to achieve a balance between structural integrity and efficient energy absorption. Further investigation is required to optimize the design for minimal deformation and maximal energy absorption efficiency.

## 5. Conclusions

This study investigated the dynamic mechanical response of lattice sandwich structures subjected to underwater shock wave loading using shock tube experiments and a 3D-DIC measurement system. An LS-DYNA finite element model of the lattice sandwich structure under underwater shock wave loading was developed and validated against experimental results. The effects of lattice relative density and target plate thickness on structural dynamic response and energy absorption efficiency were analyzed, yielding the following conclusions:

(1) Under impact loading, the deformation of a square sandwich plate initiates at the four corners and progresses inwards toward the center. The presence of the lattice core results in differing deformation rates between the face plate and back plate.

(2) Increasing relative density reduces structural deformation under constant projectile velocity. At a relative density of 0.3, the maximum back plate displacement is reduced by 22.2% compared to a relative density of 0.1. At higher projectile velocities, greater relative densities correlate with higher total energy absorption by the structure, with the lattice absorbing a larger proportion of the total energy.

(3) Variations in target plate thickness have a more pronounced effect on the face plate than the back plate. Increasing target plate thickness significantly reduces overall structural deformation. At a thickness of 1.5 mm, the maximum back plate displacement decreases sharply by 47.6%. However, energy absorption efficiency decreases by 20% due to reduced plastic deformation.

(4) The face plate and lattice primarily function as structural supports, while the back plate deformation is synergistically influenced by lattice deformation. Under low projectile velocities, excessively high target plate thickness or lattice relative density results in a significant portion of the energy being transmitted through the structure to subsequent components.

## Figures and Tables

**Table 1:** Dimensions of the lattice cores.

Relative Density *ρ*	Length *L* (mm)	Radius *R* (mm)
0.1	10	0.81
0.2	10	1.15
0.3	10	1.41

**Table 2:** Experimental results.

Case	Flyer Velocity (m/s)	Lattice Relative Density	Back Plate Peak Deflection (mm)	Peak Pressure (Mpa)
Case 1	41.58	0.1	4.222	12.417
Case 2	41.49	0.2	3.834	12.253
Case 3	41.52	0.3	2.489	12.312

**Table 3:** Fluid–solid coupling keyword setup.

Parameter	CTYPE	NQUAD	PFAC	MCOUP
Value	4	2	0.1	0

**Table 4:** 1100-O aluminum alloy material parameters.

Parameter	*ρ (*kg/m^3^)	*E* (Gpa)	*μ*	*K*	*N*
Value	2710	24.37	0.33	0.2557	0.392

**Table 5:** 4340 alloy steel and Q345 steel material parameters.

Materials	*ρ* (kg/m^3^)	*E* (Gpa)	*μ*	*σ*_y_ (Mpa)
4340 alloy steel	7850	200	0.29	792
Q345 steel	7850	211	0.3	345

**Table 6:** Equation of state parameters of water medium.

Materials	*ρ *(kg/m^3^)	*C *(m/s)	*S*_1_	*S*_2_	*S*_3_	GAMAO	*V*_0_
Water	998	1647	1.921	0.096	0	0.35	1

**Table 7:** Equation of state parameters of air medium.

Materials	*ρ* (kg/m^3^)	*C*_0_	*C*_1_	*C*_2_	*C*_3_	*C*_4_	*C*_5_	*C*_6_
Air	1.185	0	0	0	0	0.4	0.4	0

## Data Availability

The raw/processed data required to reproduce these findings cannot be shared at this time as the data also forms part of an ongoing study.
